# Evaluation of a novel forward-looking optical coherence tomography probe for endoscopic applications: an ex vivo feasibility study

**DOI:** 10.1007/s00464-024-11353-1

**Published:** 2024-11-04

**Authors:** Femke J. C. Jacobs, Vincent Groenhuis, Ibeltje M. de Jong, Iris D. Nagtegaal, Maroeska M. Rovers, Geert J. Bulte, Jurgen J. Fütterer

**Affiliations:** 1 Department of Medical Imaging, Radboud University Medical Center, Nijmegen, The Netherlands; 2 Robotics and Mechatronics, University of Twente, Enschede, The Netherlands; 3 Scinvivo B.V, Eindhoven, The Netherlands; 4 Department of Pathology, Radboud University Medical Centre, Nijmegen, The Netherlands; 5 Department of Gastroenterology and Hepatology, Radboud University Medical Center, Nijmegen, The Netherlands

**Keywords:** Colonoscopy, Gastroenterology, Colorectal lesion, IDEAL-D stage 0

## Abstract

**Background:**

As a result of recent advances in the development of small microelectromechanical system mirrors, a novel forward-looking optical coherence tomography (OCT) probe with a uniquely large field of view is being commercially developed. The aim of this study is to prospectively assess the feasibility of this advanced OCT probe in interpreting ex vivo images of colorectal polyp tissue and to identify necessary steps for further development.

**Methods:**

A total of 13 colorectal lesions from 9 patients, removed during endoscopic resection, were imaged ex vivo with the OCT device and compared with histopathological images that served as the gold standard for diagnostics. Normal tissue from one patient, removed during the endoscopic procedure, was imaged as a negative control. We assessed the presence of features indicative for polyp type and degree of dysplasia, by comparing OCT images to histopathological images and by evaluating the presence of OCT-specific features identified by previous studies, such as effacement (loss of layered tissue structure), a hyperreflective epithelial layer, and irregularity of the surface.

**Results:**

As verified by corresponding histological images, tissue structures such as blood vessels and tissue layers could be distinguished in OCT images of the normal tissue sample. Detailed structures on histological images such as crypts and cell nuclei could not be identified in the OCT images. However, we did identify OCT features specific for colorectal lesions, such as effacement and a hyperreflective epithelial layer. In general, the imaging depth was about 1 mm.

**Conclusion:**

Some relevant tissue structures could be observed in OCT images of the novel device. However, some adaptations, such as increasing imaging depth using a laser with a longer central wavelength, are required to improve its clinical value for the imaging of colorectal lesions.

**Graphical abstract:**

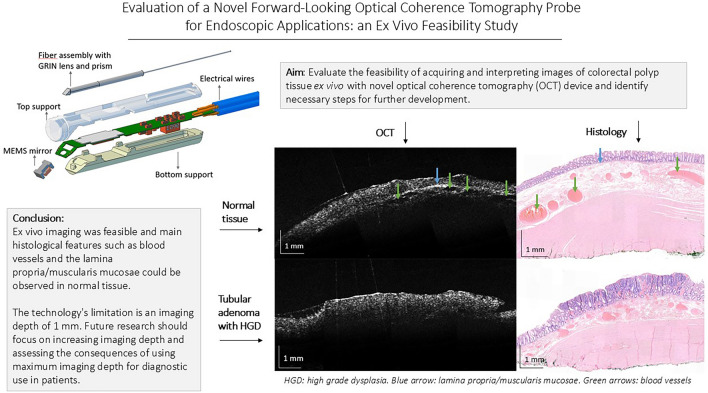

**Supplementary Information:**

The online version contains supplementary material available at 10.1007/s00464-024-11353-1.

Colonoscopy is the standard procedure for the detection of colorectal cancer and an important element of colorectal cancer screening programs worldwide [1]. The early detection of colorectal cancer and its precursor lesions in the Dutch colorectal cancer screening program has resulted in more patients with early-stage colorectal cancer and the early removal of precursor lesions (polyps) has led to a reduction in mortality caused by colorectal cancer [2]. Despite the proven value of colonoscopy in a large proportion of patients, visual inspection of endoscopic images alone is sometimes not sufficient to differentiate between benign and malignant polyps. Especially in large colorectal polyps, the sensitivity of T1 carcinoma detection has reported values as low as 20.9% up to 78.7% [3, 4]. An accurate assessment of polyp type, characteristics, and the degree of dysplasia is essential to determine the appropriate treatment strategy [5, 6]. The additional use of imaging technology that can provide detailed subsurface information might improve the endoscopic recognition of colorectal lesions.

Optical coherence tomography (OCT) is a technology that uses low coherence near-infrared light to make high-resolution cross-sectional images of tissue [7]. Studies have shown that features on OCT images can be used to distinguish between different types of colorectal polyps and different degrees of dysplasia [8–12]. In the past decennia, several OCT probe prototypes have been developed for endoscopic use. However, most of these prototypes were never developed into a commercially available device or failed once released to the market, because of technological challenges including miniaturization and compatibility with existing endoscopic equipment. Due to recent progress in the development of small microelectromechanical system (MEMS) mirrors, a novel forward-looking OCT probe for endoscopic use is being commercially developed. OCT probes can generally be divided into side-viewing and forward-looking devices [13, 14]. Forward-looking probes are more suited for quick in situ assessment of lesions identified during endoscopy, because the design allows for efficient imaging of smaller regions in the same line of sight as images from conventional forward-looking endoscopes [14–16]. The probe has an estimated lateral and axial imaging resolution of 30 µm and 6 µm, respectively, and a uniquely large field of view (imaging width of one 2D OCT image) as compared to previous forward-looking OCT probes. With a total outer diameter of 2.5 mm and a rigid length of 17 mm at the probe tip, this probe can be used as an add-on in all commercially available colonoscopes [13].

The probe is currently in an early stage of development, corresponding to stage 0 of the IDEAL-D (Idea, Development, Exploration, Assessment, and Long-term study) framework that provides recommendations for the development of medical devices [17, 18]. Stage 0 focusses on optimizing the design of the device before the first in-human study. The probe was originally developed for in vivo assessment of bladder lesions. However, to assess whether the device is applicable for other indications as well, this study focused on imaging colorectal lesions. We aim to evaluate the feasibility of acquiring and interpreting OCT images of colorectal polyp tissue ex vivo with this novel device and to identify the necessary steps for further development.

## Methods

### Patients

The study protocol was evaluated by the Medical Research Ethics Committee East-Netherlands (file number: 2021–12986), who determined that it was not subject to the Medical Research Involving Human Subjects Act. However, in the interest of maintaining complete transparency with patients, written informed consent was obtained. In total, 9 patients with 13 polyps were included. Initially, adult patients undergoing endoscopic resection for colorectal polyps of any size were included. Inclusion criteria were refined after the first two patients, when the images of small polyps removed by polypectomy appeared difficult to correlate to histopathological images, since these polyps are not systematically processed. To obtain larger samples that are systematically processed, inclusion criteria were restricted to patients with non-pedunculated polyps removed by endoscopic mucosal resection (EMR), endoscopic submucosal dissection, or endoscopic full-thickness resection (eFTR). The latter two procedures provide tissue samples that are resected en bloc and, therefore, are likely less damaged by fragmentation and coagulation. eFTR additionally results in thicker samples containing all colonic wall tissue layers and (more) normal tissue, the latter of which can be used as a negative control. Endoscopic resection was performed according to the standard of care by any gastroenterologist, but mostly by GJB. If piecemeal resection was required, it was attempted to remove the polyp in as few pieces as possible.

### OCT system

**Fig. 1:**
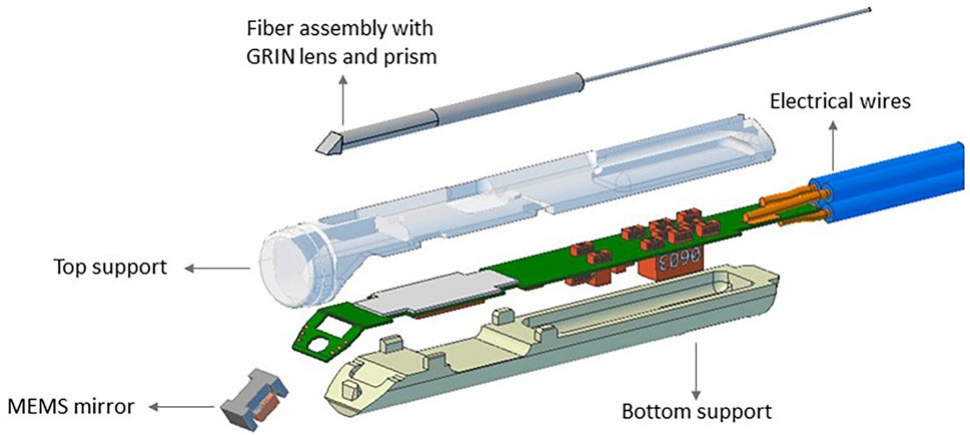
Schematic overview of catheter design. Important elements of the design are indicated

OCT is a non-invasive optical imaging technique that can capture high-resolution cross-sectional images of biological tissues based on the backscattering of light. An OCT system uses low coherence light that is being split into a reference arm and a sample arm. Sensors obtain depth resolutions by detecting an interference signal formed between the reflected light of both paths. Swept source OCT uses a near-infrared laser that sweeps around a central wavelength, where using different wavelengths allows capturing different frequency spectra. Corresponding depth profiles are reconstructed using an inverse Fourier transform. During this study, a novel MEMS-based forward-looking OCT probe (Scinvivo B.V., Eindhoven, The Netherlands) was used (Fig. 1), which allows for the fast and real-time acquisition of multiple scans in a forward-looking direction. The OCT system used in this study consisted of a near-infrared swept source laser (HSL-1, Santec Holdings Corporation, Komaki City, Aichi, Japan), an interferometer (IVS-1000, Santec Holdings Corporation, Komaki City, Aichi, Japan), and an OCT probe. The used swept source laser has a central wavelength of 1060 nm and a sweep rate of 400 kHz. Using an optical fiber, the laser is directed to a grin lens and prism in the tip of the probe to focus the laser beam. The moving MEMS mirror scans the laser beam with an optical field of view of around 20° with a frequency of 550 Hertz, resulting in a laser spot being scanned across the tissue along a short line. Each depth profile captured at a specific point along the scanned line will represent one A-scan. The complete 2D image consists of multiple A-scans next to each other and is referred to as a B-scan. Each time the MEMS mirror did a complete sweep, one B-scan is captured, meaning that the scanning rate of the OCT system is equal to the moving frequency of the mirror (i.e., 550 Hertz).

**Fig. 2:**
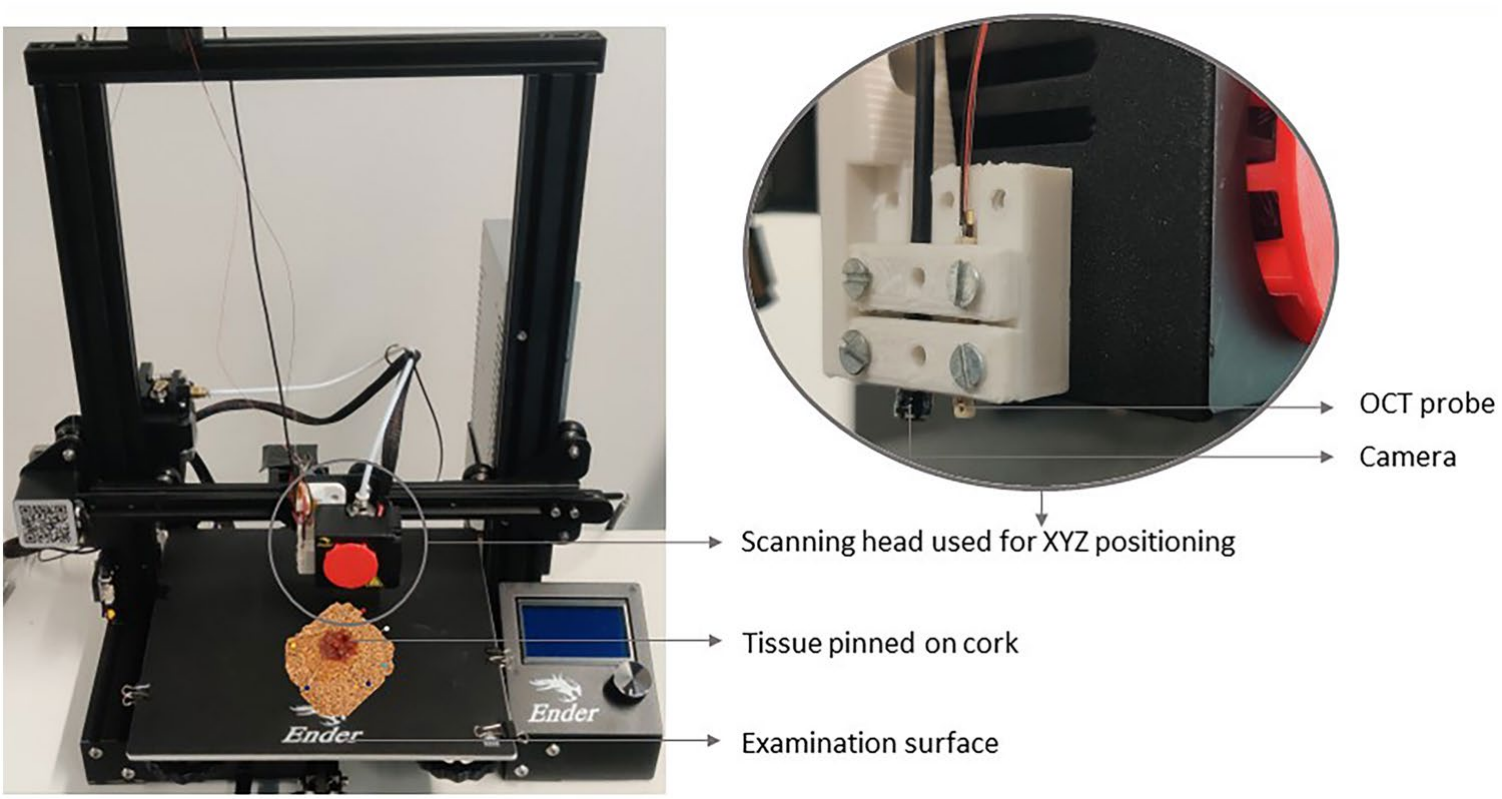
Set-up of the motorized XYZ stage that was used for stepwise and automated tissue scanning

The OCT testing station consisted of the OCT system as described above and a motorized XYZ stage to which the probe was mounted (Fig. 2). The XYZ stage was used to precisely position the normally hand-held probe in different XYZ positions to enable stepwise scanning of the tissue. It consisted of a converted 3D printer and software to control the print head. The OCT probe was attached to the scanning head, of which the movement was determined by step size, step count, and direction. The resected tissue was placed on the examination surface of the XYZ stage for scanning. To assess the scanning location of the OCT probe on the tissue, a camera was mounted next to the OCT probe on the print head (Fig. 2). The OCT as well as the camera images were displayed on a monitor and saved as raw data. The OCT system was placed on a medical car to facilitate transportation.

### OCT imaging procedure

**Fig. 3:**
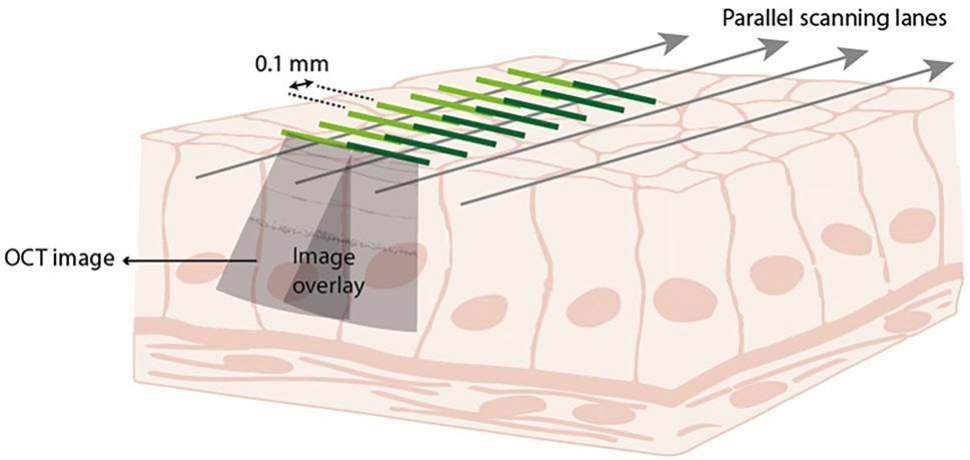
Overview of imaging procedure. Gray arrows represent the scanning lanes of the OCT catheter. Cross-sectional B-scans (light gray cone-shaped frames), were captured every 0.1 mm as depicted by the light and dark green lines. Between subsequent scanning lanes there is an overlap between OCT images, the extent of which is determined by the imaging depth, view angle, and the distance between parallel scanning lanes (Color figure online)

After resection, the tissue was immediately pinned on cork with the mucosa facing upward. When the tissue was removed en bloc, the complete sample was used for imaging. In case of piecemeal resection, the largest piece containing polyp tissue was used for imaging. Using the motorized XYZ stage set-up, the tissue was scanned in parallel lanes with an overlay of the OCT images to facilitate image stitching (Fig. 3). Within the parallel scanning lanes, OCT scans and camera images were collected every 0.1 mm. After scanning, the tissue was immediately fixed in 4% buffered formaldehyde and transported to the pathology laboratory for standard of care histological processing.

### Histological processing

During histological processing, the distribution of tissue slices over histological cassettes was documented with pictures to be able to estimate the location of the histological slices within the tissue sample. After histological processing, the hematoxylin and eosin-stained histological images were examined by a pathologist according to the standard of care.

### OCT image processing

Each time a B-scan was captured, the raw interference signal was processed with the use of the interferometer device of Santec. This signal was resampled after which a discrete inverse Fast Fourier transform was applied to determine the intensity value of the signal at different imaging depths. The S-vision software (Scinvivo B.V., Eindhoven, The Netherlands), applied an averaging step on the data for noise reduction and an interlacing step to improve lateral resolution of the OCT image.

Depending on polyp size, up to around 1000 OCT images were acquired for each sample (4–11 parallel scanning lanes, up to 150 images per lane). The OCT images contain pixel data in a cylindrical coordinate frame with the center point of the OCT images located at the position of the MEMS mirror. The data are projected onto the appropriate slices using transformations determined from the intrinsic probe parameters (field of view, axial resolution, lateral resolution) and displacements by the XYZ stage. OCT B-scans of adjacent parallel lanes within one slice partially overlap each other. Adjacent B-scans were combined for this study, to create a complete image of tissue cross-sections. Several strategies are possible to combine the overlapping data. Taking the average pixel value has the drawback that the signal-to-noise ratio in the overlapping regions visibly differs from the non-overlapping regions, so the pixel values of the nearest OCT slice were used instead. After stitching a subset of the images, the reconstructed slices were carefully examined to investigate inconsistencies in the stitching process. In particular the alignment of overlapping regions was evaluated and if necessary the inexact reconstruction parameters were iteratively tuned to improve the overlapping quality. Especially the field of view of the OCT probe and its mounting angle in the XYZ stage have shown some variability between successive scanning sessions and therefore, these may need to be re-evaluated for each reconstruction procedure for optimal stitching quality. The final image processing steps were aimed at image optimalization. Equal normalization as well as gamma correction was applied to the intensity values of all the stitched OCT image for contrast enhancement.

### Image interpretation

OCT images were interpreted by visual comparison to histological images and by visual identification of features specific for OCT images of colorectal lesions. For the visual comparison to histological images, histological images of the tissue that showed clear diagnostic features specific for the polyp type were selected by IDN (18 years of experience). These images were matched with OCT images of the corresponding location as follows. Using the camera images that were collected simultaneously with OCT images, in which the path of the laser spot could be observed, an estimation of the OCT scanning location could be made. By comparing the camera images obtained during OCT scanning with the pictures taken during histological processing, the OCT scans and histological images could be matched manually by FJCJ. Subsequently, the presence of the diagnostic features in the OCT images was described based on visual inspection by FJCJ. To identify features specific for OCT images, we assessed the presence of features specific for colorectal lesions as described in Trindade et al., including hyperreflectivity, loss of layered tissue structure (effacement), surface irregularity, and a high number of glandular structures [9]. The presence of hyperreflectivity was defined as a lighter grayscale of the epithelial layer as compared to the submucosal layer of the tissue. Effacement refers to the absence of a lamina propria/ muscularis mucosae in the OCT image that can be recognized as a highly reflective layer between the epithelial and the submucosal layer. Irregularity of the surface was identified when at least 50% of the OCT images of the lesion showed a lack of smooth surface as seen in the normal tissue example.

### Data analysis and statistics

We used descriptive statistics to present continuous variables as a median with their range. Categorical variables were presented as numbers and percentages. CaseViewer v2.4 was used to view histopathological images. S-Vision software developed by Scinvivo was used for OCT image processing. The latter is not publicly available.

## Results

A total of 9 patients with 13 colorectal polyps were imaged with the novel OCT probe. Patients had a median age of 67 (range: 40–78) and were predominantly male (66.7%) (Table 1). The consecutive inclusion of patients facilitated an iterative process of optimization, including the study workflow (e.g., improved data collection for OCT and histology image matching), the implementation of a higher-quality MEMS mirror to increase the field of view of OCT images, and updates in the imaging software. This resulted in improved matching between histology and OCT images, an increase in field of view, higher image contrast, and more accurate image stitching. Characteristics of the included patients and polyps are presented in Table 1. The largest proportion of polyps was removed using hot-snare EMR (69.2%). One polyp was removed by eFTR because of suspicion of malignancy. The tissues originated from various locations of the colon and included a large variety of polyp types, of which tubular adenomas with low-grade dysplasia (LGD) were the most abundant (38.5%). In the tissue sample removed by eFTR, a significant proportion of normal tissue was included, which was imaged with OCT as a negative control.

**Table 1:** Patient and polyp characteristics

Characteristics	Number (%)	Median (range)
Patient information (n = 9)		
Age		67 (40–78)
Gender (male)	6 (66.7)	
Polyps per patients		1 (1–2)
Colorectal polyps (n = 13)		
Diameter (n = 11)*		9 mm (2–28 mm)
Resection technique		
(Cold/hot snare) polypectomy	3 (23.1)	
Endoscopic mucosal resection	9 (69.2)	
Endoscopic full-thickness resection	1 (7.7)	
Histological diagnosis		
Hyperplastic	1 (7.7)	
Inflammatory	1 (7.7)	
SSL without dysplasia	2 (15.4)	
SSL with dysplasia	1 (7.7)	
Tubular adenoma with LGD	5 (38.5)	
Tubular adenoma with HGD	1 (7.7)	
Tubulovillous adenoma with LGD	1 (7.7)	
Traditional serrated adenoma with LGD	1 (7.7)	
Localisation		
Colon ascendens	2 (15.4)	
Colon transversum	2 (15.4)	
Flexura hepatica	1 (7.7)	
Flexura lienalis	1 (7.7)	
Colon descendens	2 (15.4)	
Sigmoid colon	3 (23.1)	
Rectum	1 (7.7)	
Unknown	1 (7.7)	

*SSL* sessile serrated lesion, *LGD* low-grade dysplasia, *HGD* high-grade dysplasia

*Polyp diameter was not reported in two cases

**Fig. 4:**
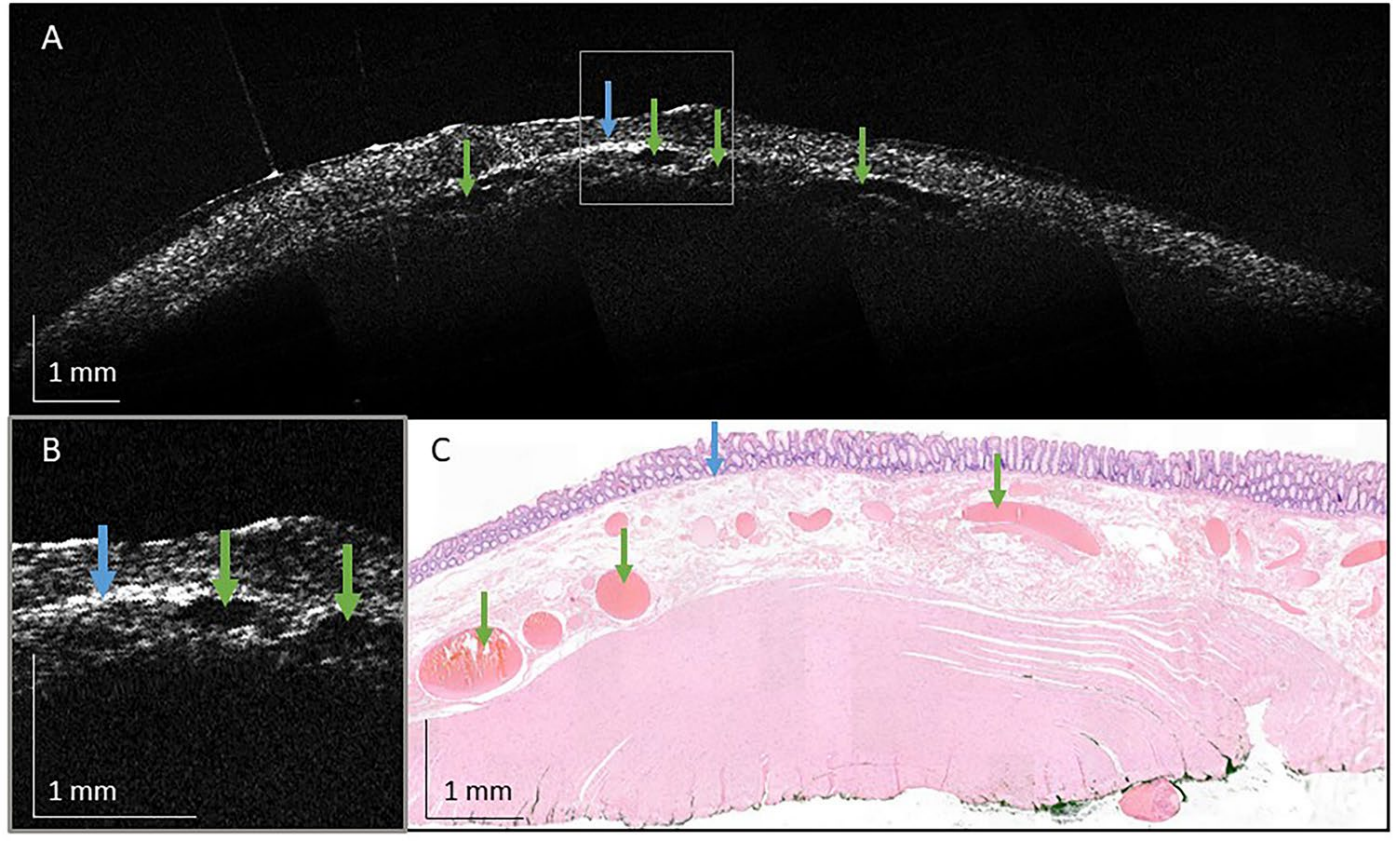
Representative images of normal colorectal tissue. The tissue was collected during endoscopic full-thickness resection of a tubular adenoma with high-grade dysplasia. **A** Representative OCT image with **B** a zoom in on the most relevant structures. **C** Corresponding histological image. Blue arrows indicate the muscularis mucosae. Green arrows indicate submucosal blood vessels (Color figure online)

**Fig. 5:**
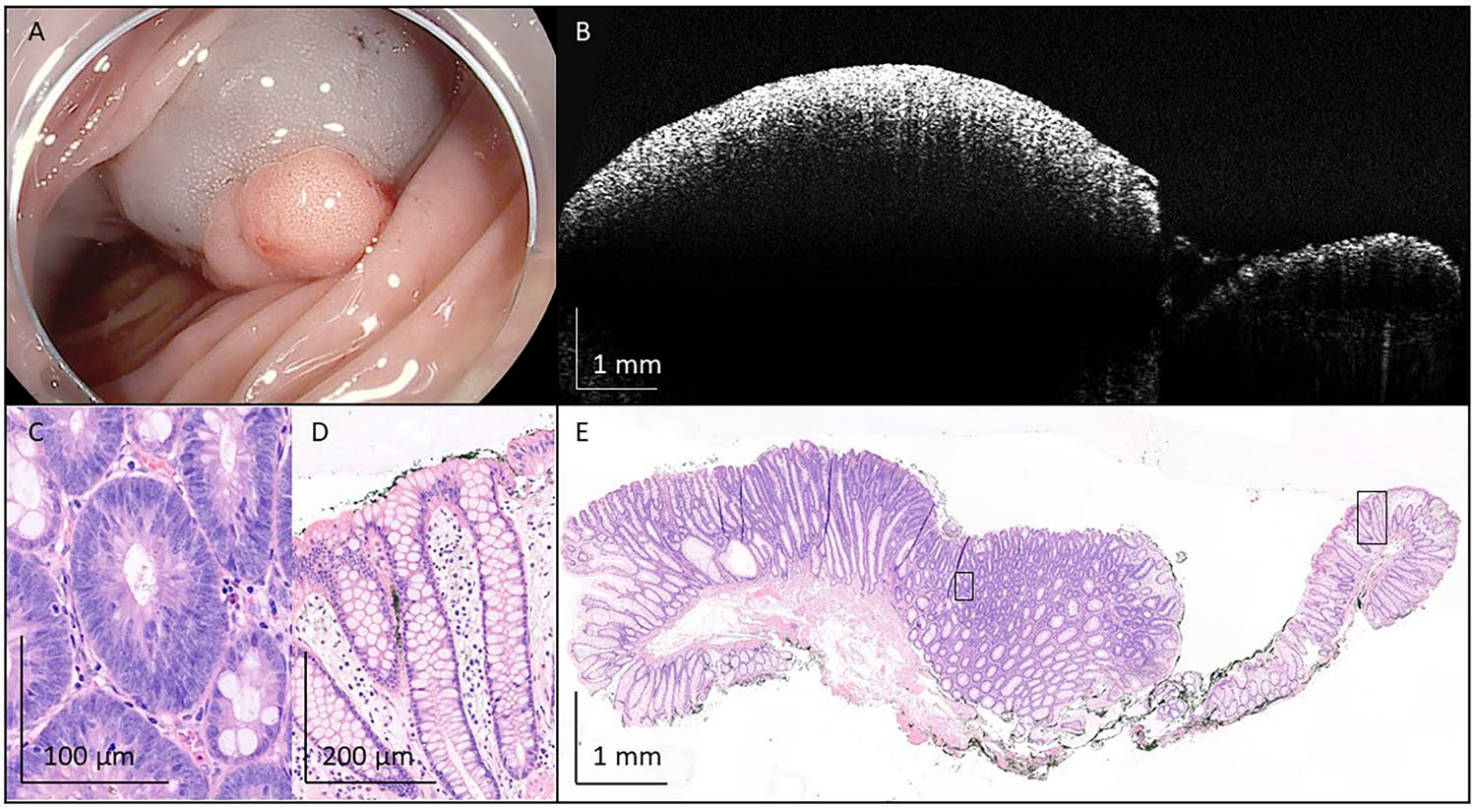
Representative image of tubular adenoma with low-grade dysplasia, removed from the flexura lienalis with endoscopic mucosal resection. **A** An endoscopic camera image before removal of the polyp. **B** A representative OCT image. **C** Crypts with pseudostratification that indicate dysplastic tissue. **D** Single cell layers that indicate normal colorectal crypts. In **E**, the full histological image is shown. Black boxes in E indicate location of C and D

**Fig. 6:**
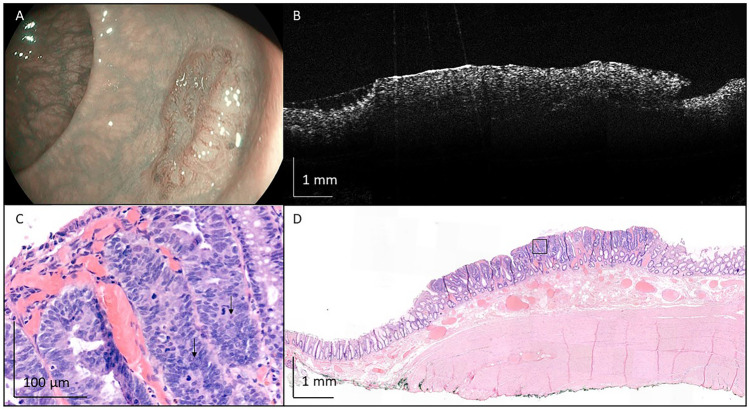
Representative image of tubular adenoma with high-grade dysplasia, removed from the sigmoid colon with endoscopic mucosal resection. **A** An endoscopic camera image before removal of the polyp. **B** A representative OCT image. **C** High-grade dysplasia, indicated by profound nuclear atypia and mitotic activity (indicated by the black arrows). In **D**, the full histological image is shown. Black box in **D** indicates location of **C**

**Fig. 7:**
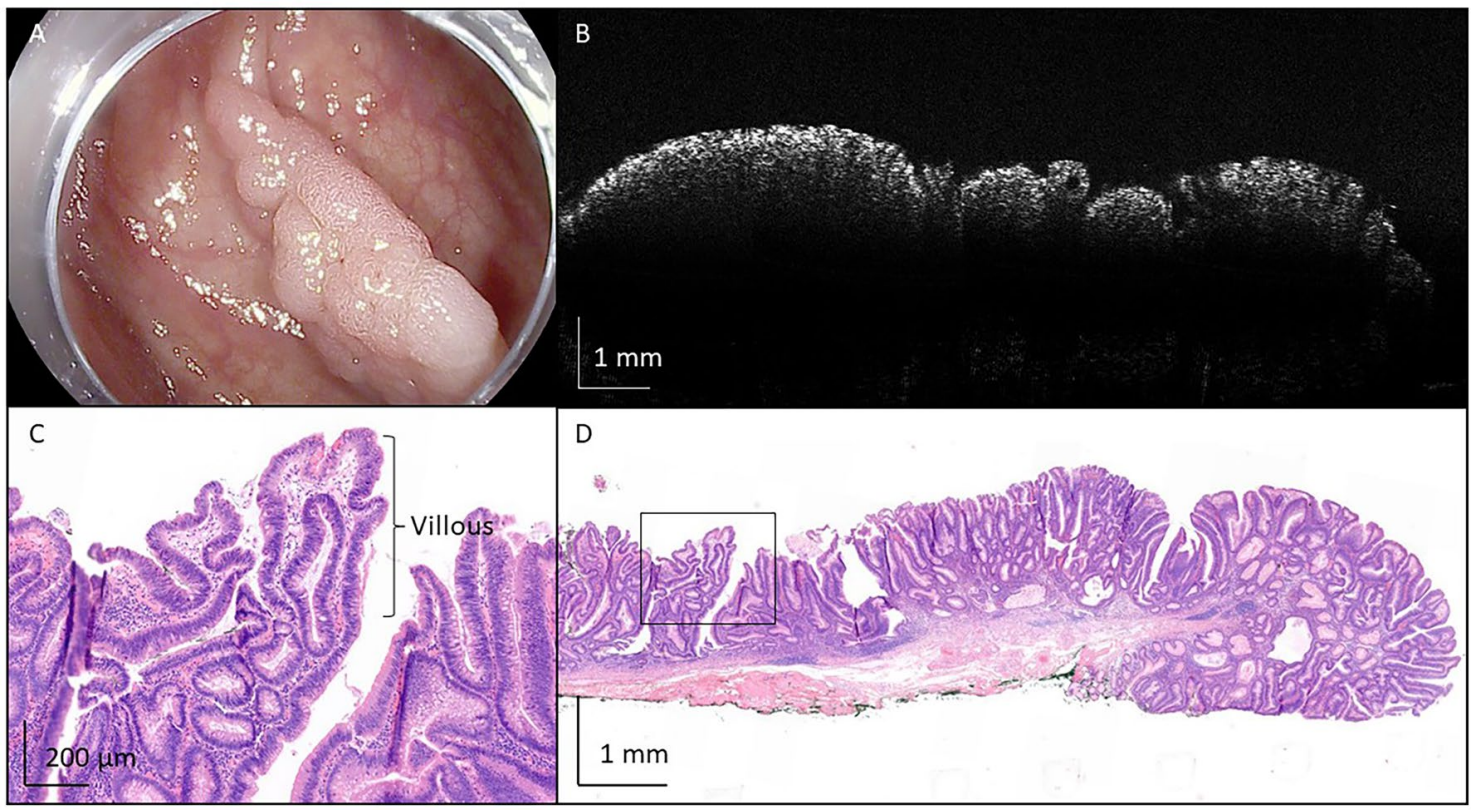
Representative image of tubulovillous adenoma with low-grade dysplasia, removed from the colon ascendens with endoscopic mucosal resection. **A** An endoscopic camera image before removal of the polyp. **B** A representative OCT image. **C** More irregular contour with formation of villi, indicative of tubulovillous adenoma. In **D**, the full histological image is shown. Black box in **D** indicates location of **C**

**Fig. 8:**
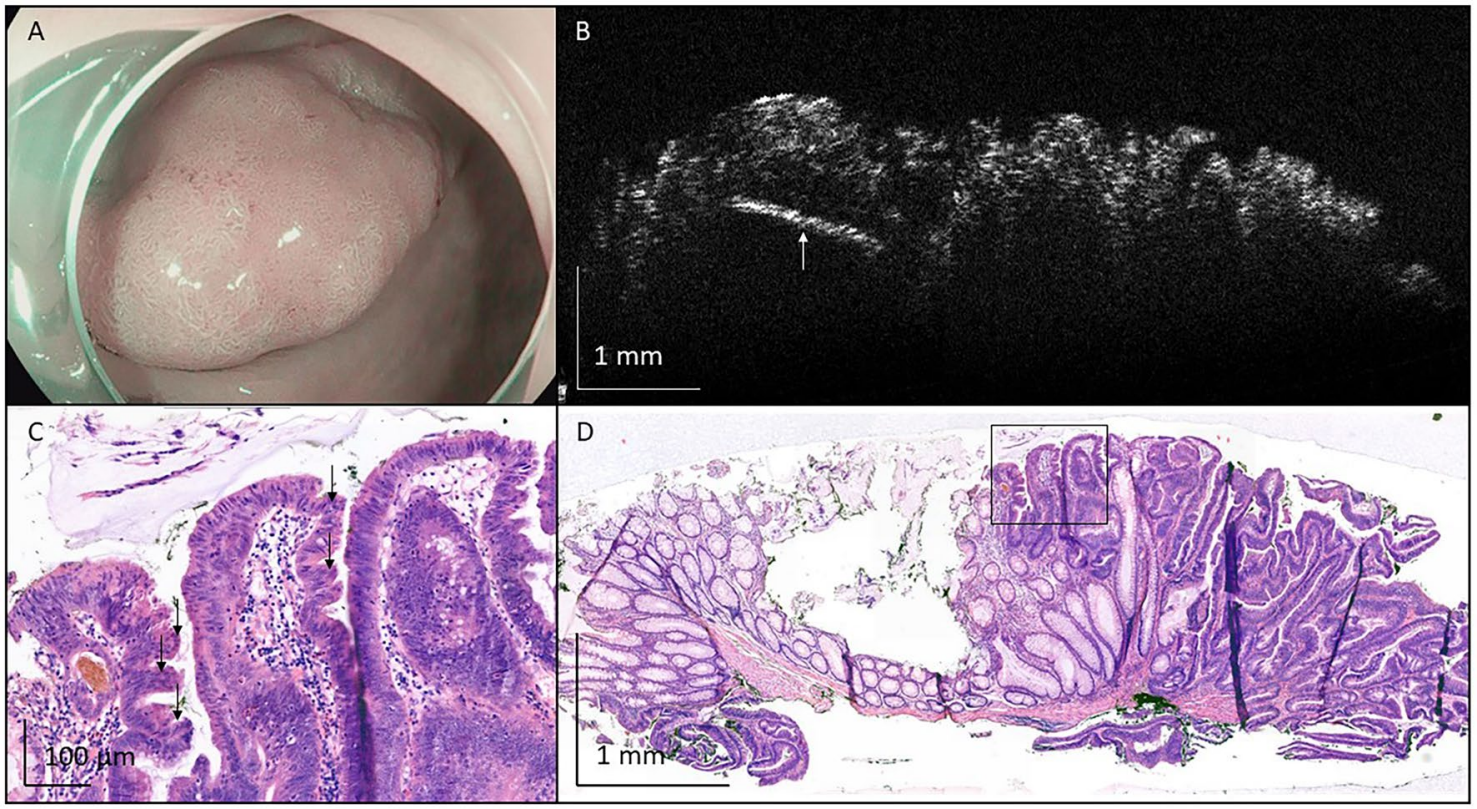
Representative image of traditional serrated adenoma with low-grade dysplasia, removed from the colon descendens with endoscopic mucosal resection. **A** An endoscopic camera image before removal of the polyp. **B** A representative OCT image. The white arrow indicates one of the pins used to attach the tissue to cork. **C** The presence of serration in crypts filled with epithelial cells with eosinophilic cytoplasm and pencil-like nuclei. In **D**, the full histological image is shown. Black box in **D** indicates location of **C**

OCT images were successfully obtained from all 13 colorectal polyps and one normal tissue sample (Figs. 4–8, Supplementary Fig. 1). OCT images of the normal tissue sample showed a layered structure in which the epithelium, the lamina propria/ muscularis mucosae, and the submucosa with clear blood vessel structures could be distinguished, as verified by corresponding histological images (Fig. 4). In terms of OCT-specific features, the tissue showed normal epithelial reflection as compared to other tissue layers and a regular surface. However, microscopic structures that are typical for normal colorectal tissue on histological images, such as epithelial crypts, could not be observed in the OCT images.

Stitched OCT images of a tubular adenoma with LGD (Fig. 5), a tubular adenoma with high-grade dysplasia (HGD) (Fig. 6), a tubulovillous adenoma with LGD (Fig. 7), and a traditional serrated adenoma with LGD (Fig. 8) show a high resemblance with correlated histological images on a tissue structure level. However, histological structures required for the diagnosis of specific polyp types were not observed on OCT images. For example, tubular adenoma with LGD is recognized based on irregular tubular crypts with pseudostratification and loss of goblet cells, compared to a single layer of cells in the crypts of normal tissue (Fig. 5C + D). In case of HGD, nuclear atypia increases with increased mitotic rates and complex architecture. (Fig. 6C). Limited presence of finger-like epithelial protrusions (up to 25% of the surface), also referred to as villi, are typical for tubulovillous adenomas (Fig. 7C), while the formation of sawtooth serrations indicates a serrated polyp (Fig. 8C). All of these structures could not be distinguished in the OCT images.

However, we did observe OCT-specific features. In all polyp OCT images, a loss of layered structure (effacement) was observed, characterized by the absence of a clear lamina propria/ muscularis mucosa. This is in contrast to the histological images, where a lamina propria and a muscularis mucosa are present. This might be explained by the increased thickness of the mucosa in polyps (Fig. 5D, 7D and 8D). The presence of a hyperreflective epithelial layer as compared to the submucosal layer was more difficult to determine. These layers could not clearly be distinguished because of effacement, which makes the interpretation of this feature subjective. Nonetheless, in the OCT images of colorectal lesions the epithelial layer seems more hyperreflective than in the normal tissue. Surface irregularity was observed in the example of the tubular adenoma with HGD, the tubulovillous adenoma with LGD, and the traditional serrated adenoma with LGD, while the tubular adenoma with LGD showed a smooth surface. No glandular structures were observed in the OCT images. The imaging depth of the cross-sectional OCT images was up to 1 mm. Hence, no information on the deeper tissue layers was captured. Moreover, since polyps were often characterized by increased thickness of the mucosa, an imaging depth up to 1 mm might be insufficient for diagnostic imaging in these polyps.

## Discussion

This pilot feasibility study revealed the technical practicality and intervention logistics of the novel forward-looking OCT probe, along with the necessary improvements to enhance its clinical relevance. We established that ex vivo imaging of endoscopically resected colorectal polyps was technically feasible. At this point the maximum imaging depth of 1 mm is the major limitation of the technology from a clinical perspective. Furthermore, we found that histological structures important for the distinguishment of polyp types could not be recognized with OCT, while the presence of OCT-specific polyp features including effacement, hyperreflectivity of the epithelial tissue layer, and irregularity of the surface could be detected [9]. However, these features are currently not sufficiently conclusive to distinguish polyp types. The results of this pilot study provide a clear future direction, emphasizing the importance of an enhanced imaging depth and the necessity of diagnostic criteria that enable the distinguishment of polyp types. In this discussion, some future directions are described.

In this study, we observed that a maximum imaging depth of 1 mm was insufficient to visualize deeper tissue layers in polyp tissue samples. It is known that pathologic conditions such as colorectal lesions can cause increased mucosal thickness [19], as was also observed in this study. This potentially explains why the presence of the lamina propria/ muscularis mucosa was only observed in OCT images of the normal tissue. Invasion depth of the lesion cannot be assessed when the lamina propria/ muscularis mucosa and deeper tissue layers cannot be visualized. Although an imaging depth of 1 mm is in line with the imaging depth observed in OCT images of colon mucosa from other studies, it seems that Trindade et al. were able to capture more detail of deeper tissue structures [9, 11, 20, 21]. First of all, Trindade et al. used a side-viewing probe that is more suited for systematic and volumetric tissue scanning and less suited for in situ assessment of lesions during colonoscopy procedures, as compared to forward-looking probes [14]. Different applications require different design choices, such as the lens type (with certain numerical aperture) or the use of other optical components (i.e., mirrors), that ultimately influence the image resolution and imaging depth. Secondly, imaging depth and resolution depend on the characteristics of the light source, alongside the optical properties of the tissue. Strong scattering and absorption reduce the achievable imaging depth in tissue, typically to around 1–2 mm. In the near-infrared wavelength spectrum suitable for optical imaging (~ 700–1600 nm), light absorption increases with wavelength, mainly due to water, and scattering strength decreases with wavelength. However, backscattering—the physical mechanism by which OCT images are formed—also decreases with wavelength. The choice of operating wavelength of an OCT system seeks to balance both factors, whereby the optimal choice depends on tissue properties (e.g., water content and backscattering properties) [22, 23]. The axial resolution that can be obtained scales with the ratio of center wavelength-squared over optical bandwidth (λ_0_^2^/Δλ) [23]. Consequently, doubling the central wavelength would require a fourfold increase in optical bandwidth in order to maintain axial resolution. The extent to which this is feasible depends on state-of-the-art in light source technology. The optical design of the probe and OCT system in this study is optimized for in vivo bladder cancer diagnostics. Since cystoscopy procedures are usually performed on a fluid-filled bladder, a shorter central wavelength (1060 nm) resulting in less absorption of the light was chosen for the initial probe design. Experimenting with different light sources with a higher wavelength could improve imaging depth.

The assessment of OCT features harbors a risk of subjective interpretation of the images. Hence, there is a demand for more objective diagnostic criteria enabling quantitative analysis. Previous studies have shown promising results with using for example the attenuation coefficient as a quantifiable criterion for diagnostic imaging with OCT [24]. Others have explored the use of deep learning techniques to support clinicians with the interpretation of OCT images [20, 25, 26]. However, both solutions would require a large set of data to recognize measurable patterns in OCT images of different tissue types, underscoring the importance of acquiring more data as a next step in the development of this device.

The major strength of this study is that it assesses the feasibility of image interpretation in an early stage of development of the novel OCT probe. This provided insight into important areas for improvement, that should be focused on in future (pre-)clinical evaluations. Some potential limitations should also be discussed. First, in this study we collected tissues removed by polypectomy or EMR. These resection techniques only remove the mucosal tissue layer with a limited amount of submucosal tissue. This was frequently done using a hot snare that results in cauterization of the tissue margin. This means that regardless of the imaging depth, imaging of the submucosal layers in these tissues was incomplete. In addition, it is uncertain whether potential tissue damage could have distorted the OCT signal and resulted in lower image quality. However, no large differences were observed with OCT images of the single eFTR sample that was less damaged and contained more tissue layers, which suggests that imaging depth is the largest limiting factor for the imaging of submucosal tissue. Second, only one sample of normal tissue was imaged, and no cancerous lesions were included in this study. For proper comparison between normal tissue, benign lesions, or malignant lesions, the inclusion of more normal tissue and cancerous lesions would be valuable. Nonetheless, this pilot feasibility study has already identified necessary technological adjustments that should be investigated and preferably implemented, before more patients are asked to participate. Third, due to instability of the MEMS mirror and the lack of a closed loop control system, variation in the field of view could be detected during tissue imaging. This also complicated the stitching of OCT images and in some cases resulted in stitched images with jagged transitions between the B-scans. A closed loop control system is currently being integrated and the stability of the MEMS mirror is being improved, leading to a more stable optical field of view. Moreover, when using the device in vivo, the static images will be replaced by real-time images with rapid acquisition times, rendering image stitching redundant.

A next step in the development of this OCT probe for imaging colorectal lesions would be to adapt the probe design for specific use in colonoscopy, including switching to a longer central wavelength. More images from normal tissue and different colorectal lesions (benign and malignant) should be acquired, which is necessary to identify additional OCT features to improve diagnostic accuracy, potentially using artificial intelligence. It would be interesting to collect tissues from patients with inflammatory bowel disease, since OCT has been suggested to be particularly valuable in these patients in which dysplasia is difficult to distinguish from surrounding inflamed tissue [27–29]. Additionally, as it is known that colonic wall thickness varies from patient to patient and by segment of the colon and depends on whether the wall is distended or collapsed [19], it is important to investigate the consequences of the maximum imaging depth (after potential adjustments) for diagnostic use in a larger patient population. Due to accessibility of the rectum for transanal endoscopic ultrasound, and the possibility to obtain deeper resection levels with techniques, such as intermuscular dissection, OCT is likely more relevant in more proximal locations. Last, usability and safety of the device should be properly tested before the first in-human pilot, for example, with a mock-up test and colorectal phantoms, respectively. Subsequently, performance and safety should be assessed in an *in vivo* clinical study. It must be investigated if it is feasible to reach and image colorectal lesions in vivo, and whether the real-time acquired images can be interpreted.

In conclusion, in this preclinical study imaging ex vivo human colorectal tissue with a novel forward-looking OCT probe, some relevant OCT-specific tissue structures could be distinguished. However, to improve the clinical value of the device specifically for the imaging of colorectal lesions, adaptations such as optimizing the central wavelength of the light source must be made.

## Supplementary Information

Below is the link to the electronic supplementary material.

Supplementary file1 (DOCX 355 KB)

## Abbreviations

EMR: Endoscopic mucosal resection
eFTR: Endoscopic full-thickness resection
HGD: High-grade dysplasia
LGD: Low-grade dysplasia
MEMS: Micro-electromechanical system
OCT: Optical coherence tomography

## References

[1] Eline HS, Arlinda R, Linda R, Robert ES, Joseph JYS, Graeme PY, Ernst JK (2015) Colorectal cancer screening: a global overview of existing programmes. Gut 64:1637 26041752 10.1136/gutjnl-2014-309086

[2] Breekveldt ECH, Lansdorp-Vogelaar I, Toes-Zoutendijk E, Spaander MCW, van Vuuren AJ, van Kemenade FJ, Ramakers CRB, Dekker E, Nagtegaal ID, Krul MF, Kok NFM, Kuhlmann KFD, Vink GR, van Leerdam ME, Elferink MAG (2022) Colorectal cancer incidence, mortality, tumour characteristics, and treatment before and after introduction of the faecal immunochemical testing-based screening programme in the Netherlands: a population-based study. Lancet Gastroenterol Hepatol 7:60–68 34822762 10.1016/S2468-1253(21)00368-X

[3] Backes Y et al (2019) Multicentre prospective evaluation of real-time optical diagnosis of T1 colorectal cancer in large non-pedunculated colorectal polyps using narrow band imaging (the OPTICAL study). Gut 68:271–279 29298873 10.1136/gutjnl-2017-314723

[4] Lonne WTM et al (2020) Optical diagnosis of T1 CRCs and treatment consequences in the Dutch CRC screening programme. Gut 69:2049 31937551 10.1136/gutjnl-2019-320403

[5] Mathews AA, Draganov PV, Yang D (2021) Endoscopic management of colorectal polyps: from benign to malignant polyps. World J Gastrointest Endosc 13:356–370 34630886 10.4253/wjge.v13.i9.356 PMC8474698

[6] Bahin FF, Heitman SJ, Rasouli KN, Mahajan H, McLeod D, Lee EYT, Williams SJ, Bourke MJ (2018) Wide-field endoscopic mucosal resection versus endoscopic submucosal dissection for laterally spreading colorectal lesions: a cost-effectiveness analysis. Gut 67:1965–1973 28988198 10.1136/gutjnl-2017-313823

[7] Huang D, Swanson EA, Lin CP, Schuman JS, Stinson WG, Chang W, Hee MR, Flotte T, Gregory K, Puliafito CA et al (1991) Optical coherence tomography. Science 254:1178–1181 1957169 10.1126/science.1957169 PMC4638169

[8] Wang C, Zhang Q, Wu X, Tang T, Liu H, Zhu SW, Gao BZ, Yuan XC (2014) Quantitative diagnosis of colorectal polyps by spectral domain optical coherence tomography. Biomed Res Int 2014:570629 24818145 10.1155/2014/570629 PMC4000955

[9] Trindade AJ, Rishi A, Hirten R, Inamdar S, Sejpal DV, Colombel J-F (2018) Identification of volumetric laser endomicroscopy features of colon polyps with histologic correlation. Gastrointest Endosc 87:1558–1564 29477303 10.1016/j.gie.2018.02.024

[10] Pfau PR, Sivak MV, Chak A, Kinnard M, Wong RCK, Isenberg GA, Izatt JA, Rollins A, Westphal V (2003) Criteria for the diagnosis of dysplasia by endoscopic optical coherence tomography. Gastrointest Endosc 58:196–202 12872085 10.1067/mge.2003.344

[11] Adler DC, Zhou C, Tsai TH, Schmitt J, Huang Q, Mashimo H, Fujimoto JG (2009) Three-dimensional endomicroscopy of the human colon using optical coherence tomography. Opt Express 17:784–796 19158891 10.1364/oe.17.000784 PMC2886288

[12] Ding Q, Deng Y, Yu X, Yuan J, Zeng Z, Mu G, Wan X, Zhang J, Zhou W, Huang L, Yao L, Gong D, Chen M, Zhu X, Liu L, Yu H (2019) Rapid, high-resolution, label-free, and 3-dimensional imaging to differentiate colorectal adenomas and non-neoplastic polyps with micro-optical coherence tomography. Clin Transl Gastroenterol 10:e00049 31192828 10.14309/ctg.0000000000000049 PMC6613865

[13] Tsai T-H, Fujimoto JG, Mashimo H (2014) Endoscopic optical coherence tomography for clinical gastroenterology. Diagnostics 4:57–93 26852678 10.3390/diagnostics4020057 PMC4665545

[14] Gora MJ, Suter MJ, Tearney GJ, Li X (2017) Endoscopic optical coherence tomography: technologies and clinical applications [Invited]. Biomed Opt Exp 8:2405–2444 10.1364/BOE.8.002405 PMC5480489 28663882

[15] Glover B, Teare J, Patel N (2020) The status of advanced imaging techniques for optical biopsy of colonic polyps. Clin Transl Gastroenterol 11:e00130 32352708 10.14309/ctg.0000000000000130 PMC7145035

[16] Liang K, Ahsen OO, Wang Z, Lee H-C, Liang W, Potsaid BM, Tsai T-H, Giacomelli MG, Jayaraman V, Mashimo H, Li X, Fujimoto JG (2017) Endoscopic forward-viewing optical coherence tomography and angiography with MHz swept source. Opt Lett 42:3193–3196 28809905 10.1364/OL.42.003193 PMC5875690

[17] Sedrakyan A, Campbell B, Merino JG, Kuntz R, Hirst A, McCulloch P (2016) IDEAL-D: a rational framework for evaluating and regulating the use of medical devices. BMJ 353:i2372 27283585 10.1136/bmj.i2372

[18] Marcus HJ, Bennett A, Chari A, Day T, Hirst A, Hughes-Hallett A, Kolias A, Kwasnicki RM, Martin J, Rovers M, Squire SE, McCulloch P (2022) IDEAL-D framework for device innovation: a consensus statement on the preclinical stage. Ann Surg 275:73–79 33856386 10.1097/SLA.0000000000004907 PMC8683254

[19] Macari M, Balthazar EJ (2001) CT of bowel wall thickening: significance and pitfalls of interpretation. AJR Am J Roentgenol 176:1105–1116 11312162 10.2214/ajr.176.5.1761105

[20] Luo H, Li S, Zeng Y, Cheema H, Otegbeye E, Ahmed S, Chapman WC Jr, Mutch M, Zhou C, Zhu Q (2022) Human colorectal cancer tissue assessment using optical coherence tomography catheter and deep learning. J Biophotonics 15:e202100349 35150067 10.1002/jbio.202100349 PMC9581715

[21] Jelvehgaran P, Alderliesten T, Georgiou G, Meijer SL, Bloemen PR, Kodach LL, van Laarhoven HWM, van Berge Henegouwen MI, Hulshof M, Rasch CRN, van Leeuwen TG, de Boer JF, de Bruin M, van Herk M (2018) Feasibility of using optical coherence tomography to detect radiation-induced fibrosis and residual cancer extent after neoadjuvant chemo-radiation therapy: an ex vivo study. Biomed Opt Express 9:4196–4216 30615728 10.1364/BOE.9.004196 PMC6157785

[22] Kodach VM, Kalkman J, Faber DJ, van Leeuwen TG (2010) Quantitative comparison of the OCT imaging depth at 1300 nm and 1600 nm. Biomed Opt Exp 1:176–185 10.1364/BOE.1.000176 PMC3005155 21258456

[23] Aumann S, Donner S, Fischer J, Müller F (2019) High Resolution Imaging in Microscopy and Ophthalmology: New Frontiers in Biomedical Optics. Chapter 3 Optical Coherence Tomography (OCT): Principle and Technical Realization, Springer, Available from: https://www.ncbi.nlm.nih.gov/books/NBK554044/32091846

[24] Chang S, Bowden AK (2019) Review of methods and applications of attenuation coefficient measurements with optical coherence tomography. J Biomed Opt 24:1–17 31520468 10.1117/1.JBO.24.9.090901 PMC6997582

[25] Saratxaga CL, Bote J, Ortega-Morán JF, Picón A, Terradillos E, del Río NA, Andraka N, Garrote E, Conde OM (2021) Characterization of optical coherence tomography images for colon lesion differentiation under deep learning. Appl Sci 11:3119

[26] Niioka H, Kume T, Kubo T, Soeda T, Watanabe M, Yamada R, Sakata Y, Miyamoto Y, Wang B, Nagahara H, Miyake J, Akasaka T, Saito Y, Uemura S (2022) Automated diagnosis of optical coherence tomography imaging on plaque vulnerability and its relation to clinical outcomes in coronary artery disease. Sci Rep 12:14067 35982217 10.1038/s41598-022-18473-5 PMC9388661

[27] Consolo P, Strangio G, Luigiano C, Giacobbe G, Pallio S, Familiari L (2008) Optical coherence tomography in inflammatory bowel disease: prospective evaluation of 35 patients. Dis Colon Rectum 51:1374–1380 18546041 10.1007/s10350-008-9304-6

[28] Shen B, Zuccaro G Jr, Gramlich TL, Gladkova N, Trolli P, Kareta M, Delaney CP, Connor JT, Lashner BA, Bevins CL, Feldchtein F, Remzi FH, Bambrick ML, Fazio VW (2004) In vivo colonoscopic optical coherence tomography for transmural inflammation in inflammatory bowel disease. Clin Gastroenterol Hepatol 2:1080–1087 15625653 10.1016/s1542-3565(04)00621-4

[29] Yuan W, Feng Y, Chen D, Gharibani P, Chen JDZ, Yu H, Li X (2022) In vivo assessment of inflammatory bowel disease in rats with ultrahigh-resolution colonoscopic OCT. Biomed Opt Exp 13:2091–2102 10.1364/BOE.453396 PMC9045891 35519259

