# Supplementary material for: Evaluation of a novel forward-looking optical coherence tomography probe for endoscopic applications: an ex vivo feasibility study
Source: Surg Endosc. 2024 Nov 4;38(12):7677–86. doi: 10.1007/s00464-024-11353-1 (PMC11615031; doi:10.1007/s00464-024-11353-1)
Supplement: Supplementary file 1 — Supplementary file1 (DOCX 355 KB) [file 464_2024_11353_MOESM1_ESM.docx]

Supplementary material


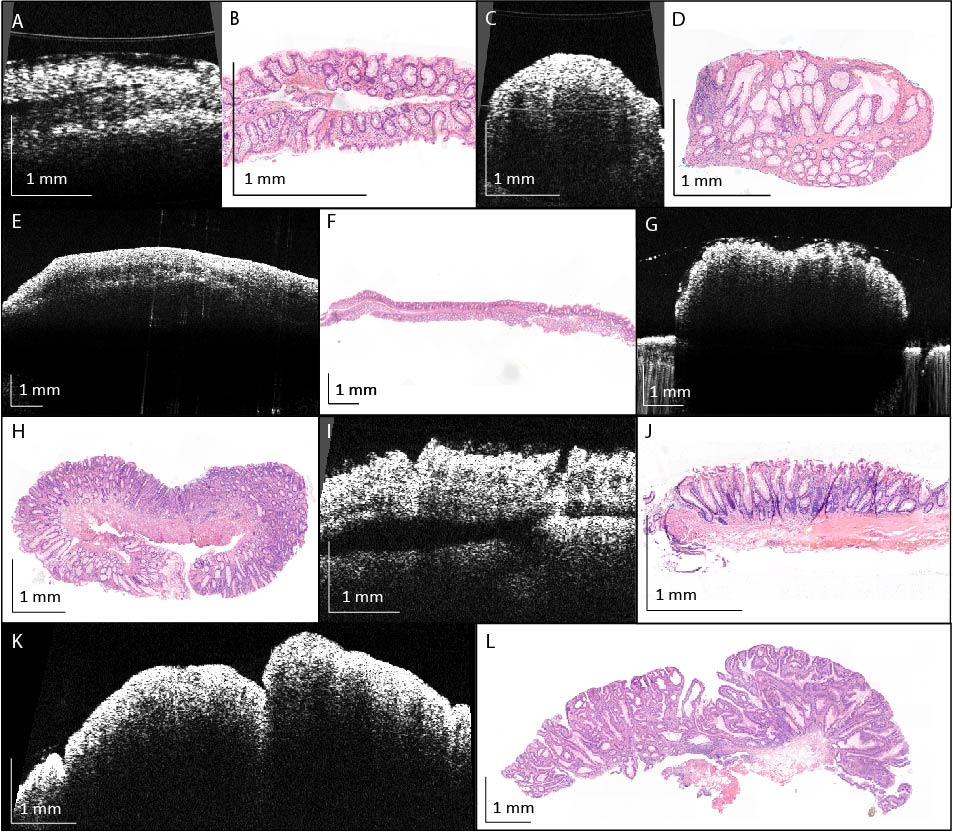


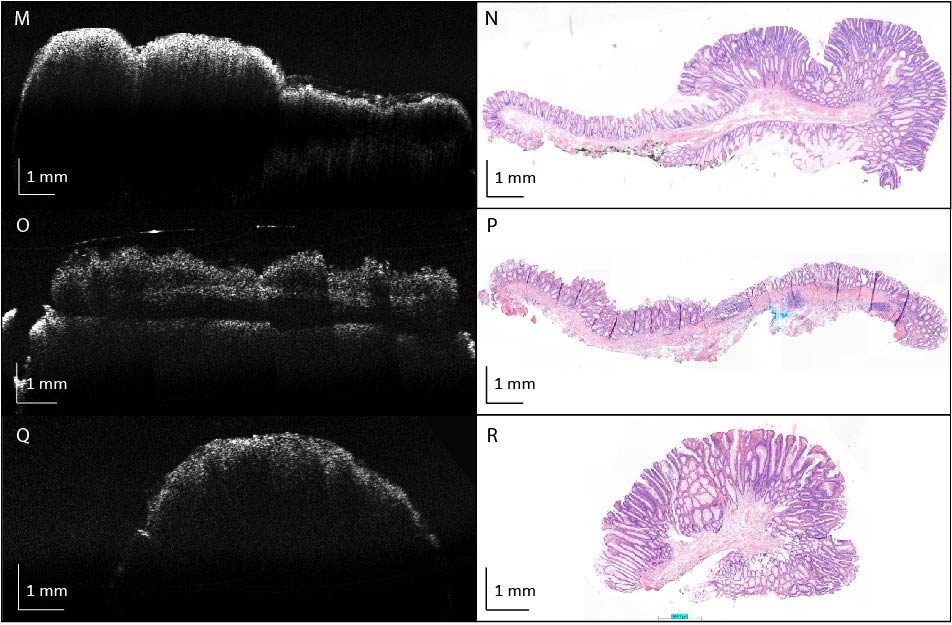


***Supplementary Figure 1****: OCT images and histopathological images of other polyps that were not used as an example in the main text. A, C, E, G, I, K, M, O and Q show OCT images of the 9 remaining polyps, while B, D, F, H, J, L, N, P and R show the corresponding histopathological images. A,B) Hyperplastic polyp. C,D) Inflammatory polyp without dysplasia. E,F, I, J) Sessile serrated lesion without dysplasia. G, H, K, L, M, N, Q, R) Tubular adenoma with low-grade dysplasia. O, P) Sessile serrated lesion with dysplasia.*

**Summary of the ‘Next gen in-vivo cancer diagnostics’ project**

To increase the survival rate and quality of life for (cancer) patients, it is crucial to make accurate diagnoses for the administration of optimal treatments. Providing the correct treatment will often lead to reduced costs due to fewer recurrences and less post-treatment care. Image-guided therapy and minimally invasive surgery will increasingly play a role in making accurate diagnoses for various oncological treatments in the coming years. One of the techniques that can be used for this purpose is Optical Coherence Tomography (OCT), which is already widely used as a scanning technique in ophthalmology. The next step for OCT is its application in vivo (within the body). However, obtaining OCT images within the body requires a catheter. A technical feasibility study for a proof-of-concept version of this catheter has already been successfully completed (MIT.Zuid.HB.672). This project aims to optimize or develop components for the catheter's clinical use.

Furthermore, the OCT catheter has thus far been explored only for the application of diagnosing and treating bladder cancer, while it could also be used for other indications within the domains of 1) inspecting resection margins of removed tissue for tumour-free status, and 2) diagnosing suspicious anomalies in the intestines and lungs. This project also investigates which other indications within these two domains are suitable for the use of the OCT catheter.

The objective of this project is to develop and validate a prototype OCT catheter system with the following characteristics:

• Provides high imaging quality with accurate image orientation, allowing for clear distinction of all tissue layers (lateral and axial resolution of at least 40 µm).

• Can precisely locate the tumour upon re-entry of the endoscope.

• Is unaffected by motion artifacts caused by the patient's breathing or heartbeat (image refresh rate above 120 Hz).

• Is robust and capable of withstanding clinical conditions.

• Yields reproducible results through automation (results independent of the surgeon and catheter renewed per procedure).

• Is safe for patients and suitable for use on the operating table (catheter is at least 1.5 m long and complies with Medical Device Regulation 2017 requirements).

• Is versatile for multiple indications, with varying catheter requirements for different indications such as catheter diameter (possibly smaller than 2.5 mm in diameter) or the medium in which the catheter can be used (air vs. water).

• Features a catheter with a short rigid segment (less than 15 mm), making it compatible with standard endoscopes.

• Is mass-produceable at a low cost (less than €200 at a production volume of at least 10,000 units per year).

The project consists of three phases: 1) the definition phase where requirements are established for the prototype OCT catheter system, 2) the development phase where innovative components are designed to enhance the proof-of-concept catheter, and 3) the clinical study wherein the prototype catheter will be tested and validated in patients.

This project involves four partners. The first is Scinvivo (SME), responsible for developing the prototype OCT catheter and parts of the prototype OCT catheter system. The second is Bruco (SME), responsible for developing the prototype ASIC, an essential catheter component. The third is Radboudumc, conducting research on potential catheter application areas, leading the valorization of the OCT catheter in tissues and subsequently in patients. The fourth partner, University of Twente, is responsible for developing imaging software and robot control.

Urologists from various Dutch hospitals have expressed interest in testing the catheter in their clinics. The medical necessity of the catheter is acknowledged by the majority of urologists (>95%). The market introduction of the OCT catheter for bladder cancer is anticipated in 2024. For other applications, it will take over a year as the catheter needs modification. The projected revenue for the OCT catheter is €27 million by 2028.

This project contributes to social sustainability by improving healthcare and fostering innovation. It leverages the innovation potential of Eastern Netherlands through collaboration between businesses (SMEs) and research institutions, capitalizing on their expertise. The project aligns with the strategic sectors of High Tech Systems & Materials (HTSM) and Health, utilizing key technologies such as ICT and manufacturing. It supports various HTSM and Health crossovers: robotics; medical imaging; smart, new materials, and the medical technology highway.
